# Misdiagnosis and Treatment of Corneal Complications Caused by Suture Exposure After Buried-Suture Double-Eyelid Blepharoplasty

**DOI:** 10.1007/s00266-023-03606-6

**Published:** 2023-08-31

**Authors:** Xin Jin, Hao Jin, Jingrao Wang, Hong Zhang

**Affiliations:** 1 Key Laboratory of Basic and Clinical Research of Heilongjiang Province, Eye Hospital, The First Affiliated Hospital of Harbin Medical University, No. 23 Youzheng Road, Harbin, Heilongjiang Province People’s Republic of China; 2 Department of Orthopaedics, The First Affiliated Hospital of Harbin Medical University, Harbin, Heilongjiang Province People’s Republic of China

**Keywords:** Misdiagnosis, Treatment, Complications, Suture exposure, Buried-suture double-eyelid blepharoplasty

## Abstract

**Purpose:**

The purpose of this study was to summarize the misdiagnosis and treatment of corneal complications associated with suture exposure in cases of buried-suture double-eyelid blepharoplasty.

**Methods:**

This study retrospectively analyzed 14 patients with palpebral conjunctival and corneal complications due to suture exposure after buried-suture double-eyelid blepharoplasty at the First Affiliated Hospital of Harbin Medical University from January 2020 to July 2022. The patients’ clinical symptoms included photophobia, lacrimation, pain, foreign body sensation, swelling of the eyelids, conjunctival hyperemia, secretion, etc. We recorded the patient's sex, age, surgical method, length of exposed suture, suture type, number of double-eyelid surgeries, surgical site, timepoint when eye discomfort occurred, misdiagnosed disease and treatment.

**Results:**

Three patients were misdiagnosed with dry eye, nine patients were misdiagnosed with viral keratitis, and two patients were misdiagnosed with allergic conjunctivitis. All 14 patients had manifestations of photophobia, lacrimation, pain, foreign body sensation and conjunctival hyperemia. Eight patients had manifestations of swelling of the eyelids. Five patients had manifestations of eye secretions. There were 8 patients with corneal epithelial injuries and 6 patients with corneal ulcers. All patients underwent suture removal without further progression. Ten patients were treated with artificial tears, and 4 patients were treated with calf serum deproteinized gel after suture removal.

**Conclusion:**

If there is postoperative eye discomfort caused by eyelid and corneal complications in patients after buried-suture double-eyelid blepharoplasty, clinicians should carefully check whether there is suture exposure and determine the cause in a timely manner. Suture removal is the best way to treat this complication.

**Level of Evidence IV:**

This journal requires that authors assign a level of evidence to each article. For a full description of these Evidence-Based Medicine ratings, please refer to the Table of Contents or the online Instructions to Authors www.springer.com/00266.

## Introduction

Noninfectious corneal ulcer is a relatively common blinding eye disease that can occur alone or can be caused by other autoimmune diseases or collagen diseases [1]. Ocular surface tissue damage factors, such as sexual epithelial defects, can also cause corneal dissolution and corneal ulceration. The disease is prone to recurring attacks and may lead to blindness if not treated in a timely manner; moreover, its clinical treatment is quite difficult [2]. Corneal ulcers caused by exposed sutures after ophthalmic surgery are often overlooked, especially in corneal transplants, corneal lacerations, and suture exposure after buried-suture double-eyelid blepharoplasty [3–5].

The double-eyelid rate of East Asians is only 50%, and consequently, double blepharoplasty is one of the most common plastic surgery operations in China [6]. The advantages of buried-suture double-eyelid blepharoplasty are (1) less trauma, less damage to blood vessels and lymphatic drainage, less postoperative swelling and a shorter recovery period; (2) a less obvious postoperative scar; and (3) a technique that is reversible and easy to repair if the postoperative effect is not good or if the double-eyelid shape is unsatisfactory. However, there are also many possible disadvantages, such as (1) the double-eyelid line easily becomes shallow and can partially or completely disappear; (2) suture implantation may cause complications such as a foreign body reaction; (3) there is an asymmetric double-eyelid line; and (4) the eyelids droop [7]. Among these complications, suture exposure or rupture is one of the causes of sterile corneal ulcers, which are easily misdiagnosed.

Patients with suture-related complications after double-eyelid blepharoplasty may have long-term chronic pain, uncomfortable traction, and the formation of upper eyelid skin nodules [8]. The nodules can be classified into two types: exposed nodules due to superficial burial of thread knots or beaded granulomas due to chronic inflammation. In addition, penetrating tarsal fixation can lead to certain complications, such as corneal irritation, which are associated with symptoms that include discomfort, a gritty sensation, and increased ocular discharge [9]. In some patients, severe corneal irritation may suddenly occur several years after surgery, and this is caused by delayed suture rupture or exposure. However, because the upper fornix conjunctiva is pulled by the double-eyelid suture, a scar is formed, and it is difficult to completely expose the fornix conjunctiva by turning the upper eyelid by hand. Clinicians may miss the hidden suture during diagnosis and treatment without finding the real cause of the corneal injury.

Therefore, this study aimed to collect the clinical manifestations, sex, age, surgical method, exposed length of sutures, number of double-eyelid surgeries, surgical site, number of visits before finding the real cause, use or nonuse of eyelid retractors, misdiagnosed diseases and treatment methods to reduce the misdiagnosis rate.

## Materials and Methods

### Participants

This study retrospectively analyzed 14 patients with palpebral conjunctival and corneal complications due to suture exposure after buried-suture double-eyelid blepharoplasty at the First Affiliated Hospital of Harbin Medical University from January 2020 to July 2022. Of these, 13 patients were female, and only one patient was male. All patients gave informed consent to the study content. The inclusion criteria were as follows: (1) buried-suture double-eyelid blepharoplasty; (2) suture nodules or exposure; and (3) corneal complications. The exclusion criteria were corneal ulcers from other causes.

### Corneal Fluorescein Sodium Staining

The stained portion of a fluorescein sodium ophthalmic test paper was dampened with 1–2 drops of sterile saline, the wet part was gently applied to the conjunctiva of the lower eyelid of the patient, and the patient was asked to blink more than 3 times until the coating was uniform. The degree of corneal injury was observed under a slit lamp cobalt blue filter.

### The Conjunctiva of the Upper Eyelid was Observed by Turning the Eyelid

The patient was instructed to look downward with both eyes and gently turn the upper eyelid. For those who failed to fully expose the conjunctiva of the fornix by flipping the upper eyelid, an eyelid retractor was used to lift the upper eyelid and observe whether there were sutures exposed.

### The Clinical Manifestations and Treatment Process of the Patients were Recorded

We recorded the patients’ clinical symptoms, including photophobia, lacrimation, pain, foreign body sensation, swelling of the eyelids, conjunctival hyperemia, and secretion, and recorded the patient's sex, age, method of operation, length of suture exposed, number of double-eyelid surgeries, surgical site, eye discomfort, number of doctor visits prior to finding the real cause, use or nonuse of an eyelid retractor, the misdiagnosed disease and the treatment.

### Suture Removal

First, the eyelid skin was disinfected and anesthetized, then the upper eyelid was flipped over (with use of an eyelid retractor in some cases) to find the exposed part of the suture, and then the whole sutures were removed.

## Results

**Fig. 1:**
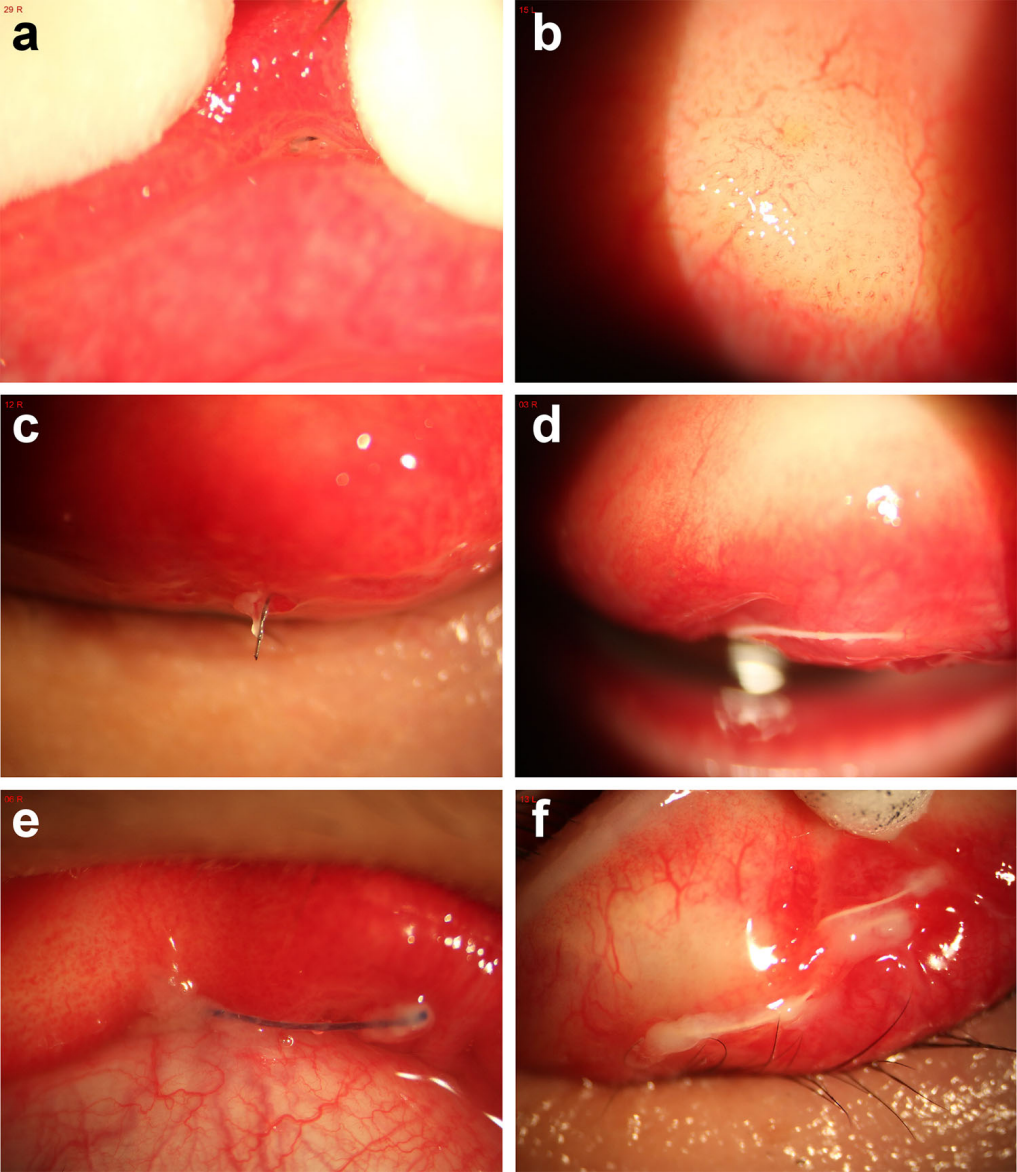
Varying degrees of suture erosion or exposure. **a** Exposed sutures of <1 mm; **b** Suture erosion; **c** Exposed sutures of 1–5 mm;** d**–**f** exposed sutures of > 5 mm

Among the 14 patients, 13 were female, and 1 was male, with ages ranging from 17 to 45 years (Table 1). All patients underwent buried-suture double-eyelid blepharoplasty. Among them, 6 patients underwent interrupted suturing, and 8 patients underwent continuous suturing (Table 1). Considering the difference in irritation symptoms in patients with exposed sutures of different lengths, we determined the number of patients with < 1 mm (Fig. 1a), 1–5 mm (Fig. 1c) and > 5 mm (Fig. 1d–f) sutures, and the results showed that there were 2 patients with <1 mm, 10 patients with 1–5 mm and 2 patients with >5 mm sutures. Most of the patients only received one double-eyelid surgery (12/14); the patients’ surgeries mainly occurred in eye clinics (5/14) and beauty studios (6/14). The patients also differed in the duration of eye discomfort after surgery: 2 patients had durations of <1 month, 2 patients had durations of 1–3 months, 4 patients had durations of 3 months–1 year, and 1 patient had a duration of >1 year. This disease is relatively easy to misdiagnose; we used eyelid retractors to find the true cause in most patients (12/14), and many patients had presented for two or more ophthalmic visits before discovering the true cause. In this retrospective analysis, the true cause of corneal complications was found in most patients (8/14) after more than three visits: three patients had been misdiagnosed with dry eye, nine patients had been misdiagnosed with viral keratitis, and two patients had been misdiagnosed with allergic conjunctivitis (Table 1).

**Table 1:** Clinical data of patients

		Number of patients
Sex	Female	13
	Male	1
Age	< 18	1
	18–25	4
	25–45	9
Method of operation	Interrupted suture	6
	Continuous suture	8
Length of suture exposed	<1 mm	2
	1–5 mm	10
	>5mm	2
Suture type	Monofilament nylon wire	12
	Multiple silk threads	2
Number of double eyelid surgeries	Once	12
	More than once	2
Surgical site	Eye clinic	5
	Beauty studio	6
	General hospital (level II)	3
When the eye discomfort occurred (postoperative)	<1 month	2
	1–3 month	2
	3 Months–1 year	4
	>1 Year	6
Number of doctors’visits prior to finding	2 Visits	3
The real cause	3 visits	3
	More than 3 visits	8
Whether to use an eyelid retractor	Yes	12
	No	2
Misdiagnosed	Dry eye	3
	Viral keratitis	9
	Allergic conjunctivitis	2

**Fig. 2:**
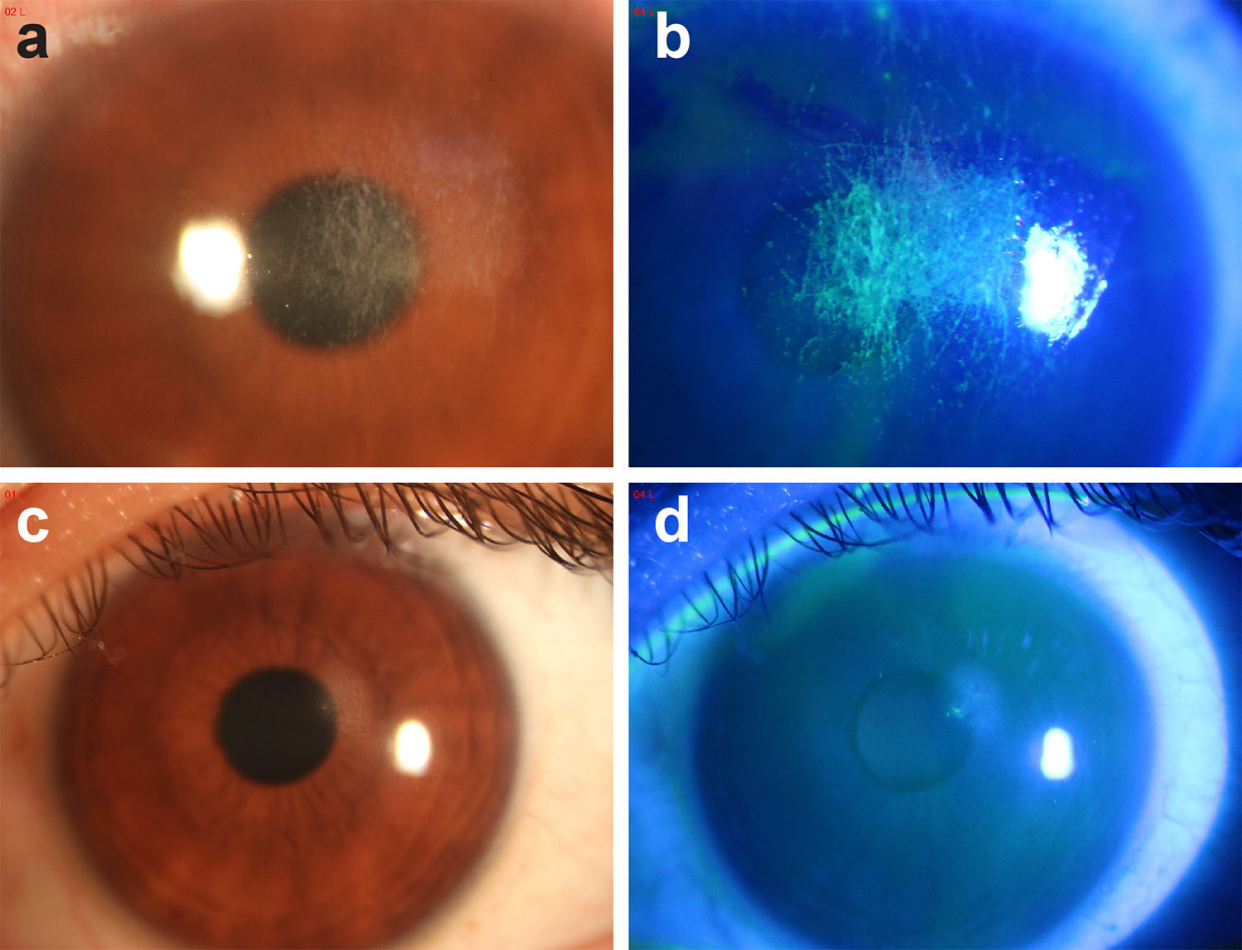
**a** Many linear lesions of varying lengths were discovered on the cornea and were partially overlapping. **b** The line structure of the corneal injuries was clearer after fluorescence staining. **c** and **d** After suture removal and artificial tears, the corneal epithelium had healed, but corneal clouding remained

**Fig. 3:**
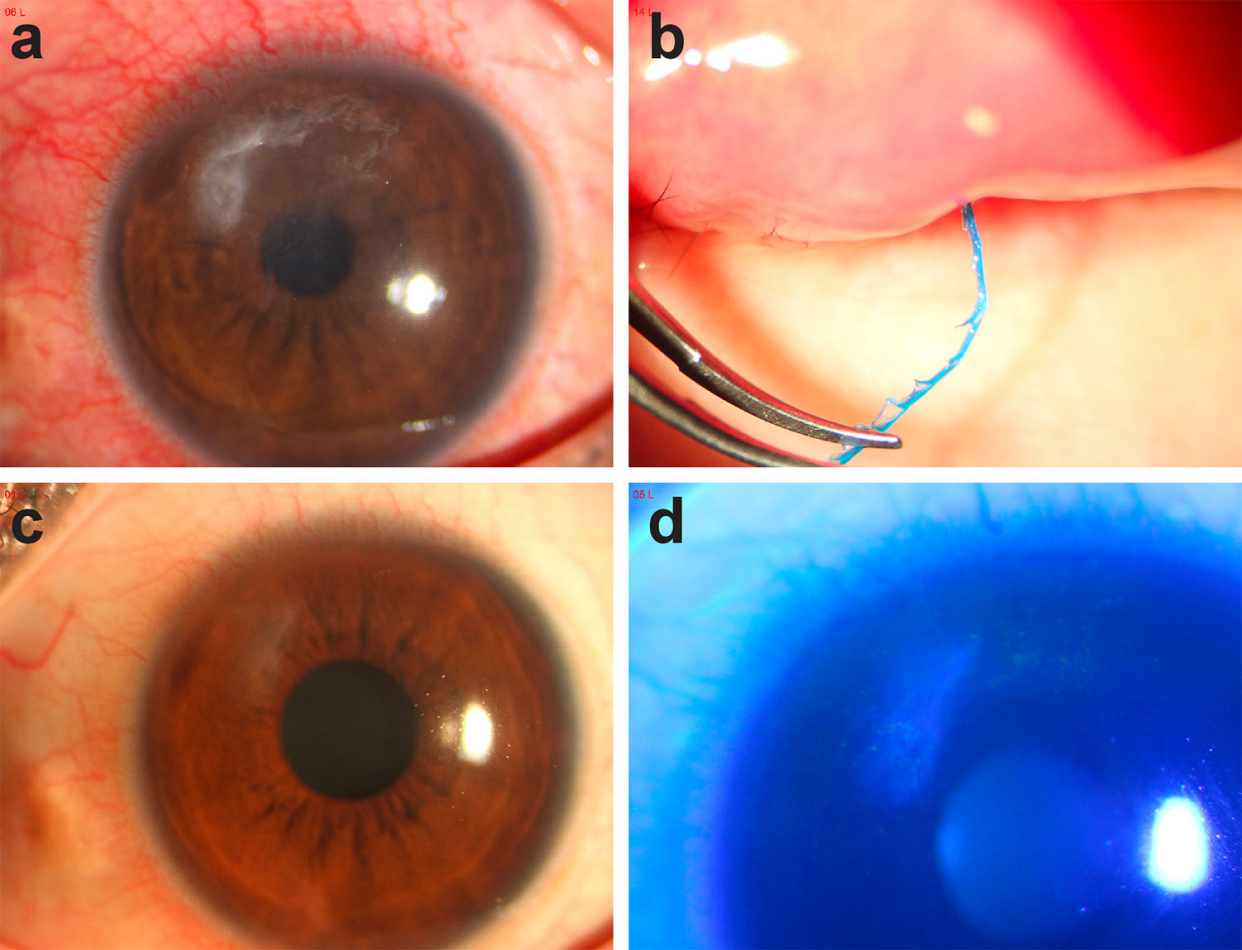
**a** An irregular ulcer with matrix edema was seen above the nasal side of the left eye. **b** A polyfilament with a length of more than 5 mm with burrs was observed when the upper eyelid was flipped over. **c** and **d** The cornea became cloudy after treatment

All 14 patients had manifestations of photophobia, lacrimation, pain, foreign body sensation and conjunctival hyperemia. Eight patients had manifestations of eyelid swelling. Five patients had manifestations of secretions. There were 8 patients with corneal epithelial injuries (Fig. 2a) and 6 patients with corneal ulcers (Fig. 3a). All patients underwent suture removal without further progression (Figs. 2c and d, 3c and d). Ten patients were treated with artificial tears, and 4 patients were treated with calf serum deproteinized gel after suture removal (Table 2).

**Table 2:** Clinical manifestations and treatment of patients

	Number of patients
Photophobia	14
Lacrimation	14
Pain	14
Foreign body sensation	14
Swelling of the eyelids	8
Conjunctival hyperemia	14
Secretion	5
Corneal epithelial damage	8
Corneal ulcer	6
Vision loss	11
Remove sutures	14
Artificial tears	10
Deproteinized calf blood extract eye gel	4

## Discussion

There are many reports of postoperative complications of buried-suture double-eyelid blepharoplasty but few reports of suture-related complications. Due to the hidden location, many doctors may not have found the suture knot hidden in the palpebral conjunctiva when the patient was first diagnosed, and the patient’s corneal-related complications may have been misdiagnosed as viral keratitis and dry eye. In some patients, the sutures were not exposed, but the knots repeatedly stimulated the palpebral conjunctiva to form nodules that caused eye discomfort.

The symptoms caused by exposure to sutures of different lengths may have different manifestations. Our study revealed that when the exposed sutures were less than 1 mm, the corneal fluorescence staining of the patients showed mostly linear staining, and the lesions could reach the superficial stromal layer. However, sutures at a length of 1–5 and > 5 mm were able to form corneal ulcers, and the linear staining was not significant. Patients with exposed sutures of < 1 mm in length had more severe corneal irritation and may have visited a doctor several times in a short period, and fewer visits were made before the cause was identified.

A 6-0 or 7-0 translucent monofilament nylon suture is used in buried-suture double-eyelid blepharoplasty. The surface of the monofilament nylon wire is smooth; there is little resistance when it is passed through tissues, and it causes little damage to tissues and is easier to hide and fix in the tissues [10]. However, due to its low friction coefficient, there is a relatively low degree of firmness of the line knots formed. However, there were still two patients in the eye clinic who were treated with multiple silk threads. In addition, the complex structure of the polyfilaments has poor resistance to bacteria and a high probability of infection. Consequently, it is necessary for patients to undergo surgery at regular medical institutions.

Furthermore, all 14 patients had manifestations of photophobia, excessive tears, pain, foreign body sensation and conjunctival hyperemia, which are symptoms similar to the those of infectious keratitis. Our study is consistent with that of Wang et al. [11]. We found that 14 patients presented for two or more visits before suture exposure was discovered, which suggested to our clinicians that if a patient has palpebral conjunctival or corneal disease and has undergone buried-suture double-eyelid blepharoplasty, we must carefully check whether there is suture erosion or exposure on the conjunctival surface of the upper eyelid; eyelid retractors should be used, if necessary, to completely flip the eyelid, find the cause in time, and protect the patient's visual function.

Suture-related complications of double-eyelid blepharoplasty must be treated by suture removal [12]. All patients in our study underwent suture removal treatment, and all patients had double-eyelid structures that remained after suture removal. All patients were given gatifloxacin ophthalmic gel (Manufactured by Xingqi Pharmaceutical) once after suture removal, and we administered artificial tears or a calf serum deproteinized gel treatment to the patients based on the depth of the ulcer. We found that if the lesions involved the superficial and middle stromal layers, the lesions healed faster with calf serum deproteinized gel.

In our study, 11 patients had a corrected visual acuity of less than 1.0 after the corneal injury had healed. After excluding lens and fundus diseases affecting vision, the visual acuity of these patients was related to corneal clouding or a nebula covering the pupillary area. This reminds us of the importance of early detection and appropriate treatment. In addition, it is worth noting that the sutures should be completely buried in the eyelid, and the conjunctival surface of the tarsal plate should be kept intact in buried-suture double-eyelid blepharoplasty to prevent eye complications caused by suture exposure [5]. Finally, we remind ophthalmologists once again that if the patient has a surgical history of double-eyelid surgery with buried sutures and presents with corneal injury or conjunctival swelling, we should consider the factor of suture exposure, carefully flip the upper eyelid to check for easily overlooked suture knots, and apply eyelid retractors if necessary.

## Conclusions

If there is postoperative eye discomfort caused by eyelid and corneal complications in patients after buried-suture double-eyelid blepharoplasty, clinicians should carefully check whether there is suture exposure and determine the cause in a timely manner. Suture removal is the best way to treat this complication.
